# High diet quality indices associated with lower risk of lipid profile abnormalities in Taiwanese kidney transplant recipients

**DOI:** 10.1038/s41598-023-46736-2

**Published:** 2023-11-11

**Authors:** I-Hsin Lin, Tuyen Van Duong, Shih-Wei Nien, I-Hsin Tseng, Yi-Ming Wu, Yang-Jen Chiang, Hsu-Han Wang, Chia-Yu Chiang, Ming-Hsu Wang, Chia-hui Chiu, Ying-Tsen Lin, Te-Chih Wong

**Affiliations:** 1 Department of Medical Nutrition Therapy, Linkou Chang Gung Memorial Hospital, Taoyüan, Taiwan, ROC; 2 School of Nutrition and Health Sciences, College of Nutrition, Taipei Medical University, Taipei, Taiwan, ROC; 3 Department of Urology, Linkou Chang Gung Memorial Hospital, Taoyüan, Taiwan, ROC; 4 Department of Medicine, Chang Gung University, Taoyüan, Taiwan, ROC; 5 Department of Business Administration, College of Management, National Changhua University of Education, Changhua, Taiwan, ROC; 6 Center for General Education, Taipei Medical University, Taipei, Taiwan, ROC; 7 Department of Health Promotion and Health Education, National Taiwan Normal University, Taipei, Taiwan, ROC; 8 Department of Nutrition and Health Sciences, Chinese Culture University, Taipei, Taiwan, ROC

**Keywords:** Biomarkers, Cardiology, Diseases, Health care, Medical research, Nephrology, Urology

## Abstract

Cardiovascular disease (CVD) and its risk factors seem to be linked with deteriorated graft function and persists as the major cause of mortality in kidney transplant recipients (KTRs). Diet quality is associated with CVD prevention in the healthy population, however, less study focuses on KTRs. The study aimed to determine the association between diet quality indices and lipid profile abnormalities as risk factors for CVD in KTRs. This prospective study enrolled 106 KTRs who had functioning allografts from September 2016. Lipid profiles included low-density lipoprotein cholesterol (LDL-C), high-density lipoprotein cholesterol (HDL-C), total cholesterol (TC), and triglyceride (TG) and were based on the National Cholesterol Education Program Adult Treatment Panel III recommendations. Three-day dietary data were collected by a well-trained registered dietitian. The Alternative Healthy Eating Index-Taiwan (AHEI-Taiwan), Alternative Healthy Eating Index-2010 (AHEI-2010), and Healthy Eating Index-2015 (HEI-2015) scores were calculated and divided into quartiles and compared accordingly. KTRs’ mean LDL-C, HDL-C, TC, and TG levels were 119.8 ± 36.6 mg/dL, 52.0 ± 17.9 mg/dL, 205.8 ± 43.9 mg/dL, and 160.2 ± 121.6 mg/dL, respectively. Compared with the lowest quartile, only the highest quartile of AHEI-Taiwan had lower TC and LDL-C levels. After adjustment for age, gender, energy, Charlson comorbidity index, transplant duration, and dialysis duration, logistic regression analysis revealed that the highest quartile of AHEI-Taiwan had 82% (odds ratio [OR], 0.18; 95% confidence interval [CI] 0.04–0.72, *p* < 0.05) lower odds of high TC and 88% (OR 0.12; 95% CI 0.03–0.58, *p* < 0.05) lower odds of high LDL-C, and the highest quartile of HEI-2015 had 77% (OR 0.23; 95% CI 0.05–0.95, *p* < 0.05) lower odds of high LDL-C. Higher adherence to a healthy diet as per AHEI-Taiwan and HEI-2015 guidelines associated with lower risk of lipid profile abnormalities in KTRs.

## Introduction

Cardiovascular disease (CVD) and its risk factors were linked with deteriorated outcomes and increased mortality and persists as the major cause of mortality rates in patients with kidney transplant^1^. Kidney transplantation recipients (KTRs) with lower immunity remain at a higher risk of CVD, due to end-stage renal disease, and renal replacement therapy may itself contribute to CVD risk; after transplantation, cardiometabolic risk factors, such as abnormal lipid profiles, may further contribute to CVD risk^2^.

Various pathogenic mechanisms have been proposed for the development of lipid profile abnormalities after kidney transplantation, including low physical activity and eating disorders; KTRs with successful transplantation can consider themselves to be free of pre-transplant dietetic restrictions and rectified kidney function from uremia can improve appetite and exceed in liberalizing the diet^3,4^. Excessive dietary fat intake, especially trans fats and saturated fatty acids, commonly increases the risk of dyslipidemia in the healthy population^5^ and KTRs^6^.

Abnormal lipid profiles result in a high incidence of CVD^7^. However, dietary intake can improve lipid profile abnormalities and prevent CVD development^8^. Many diet quality indices, including the Alternative Healthy Eating Index (AHEI) and Healthy Eating Index (HEI), have been developed and validated, and they reflect dietary food and nutrient intake and are related to CVD prevention in the healthy population^9,10^. Because of the complexity of diets, diet quality indices can serve as powerful rapid dietary assessment for medical nutrition therapy in patients with chronic disease^8^. Both HEI and AHEI are based on Dietary Guidelines for Americans and are the most commonly used indices for assessing dietary food and nutrient intake in patients with chronic disease. AHEI-Taiwan is a revised version of the AHEI that was developed according to Taiwan’s dietary recommendations^11^. Relevant studies regarding to dietary quality (especially AHEI-Taiwan) and chronic disease in Taiwanese KTRs were limited. In this study, we investigated the association between different dietary indices and lipid profile abnormalities as risk factors for CVD in Taiwanese KTRs. We hypothesized that a healthy diet quality as higher dietary index scores are associated with a lower risk of lipid profile abnormalities as risk factors for CVD in KTRs.

## Materials and methods

### Study design and participants’ enrollment

In this prospective study, we recruited 106 KTRs who is more than 18 years old and had functioning allografts without any immune-rejection in the past 3 months in Linkou Chang Gung Memorial Hospital from September 2016. We excluded 4 patients with extreme energy intake (> 3500 kcal or < 800 kcal), amputation, pregnancy, and cancer as previously described^6^. Informed consent was obtained from each participant before the interview. All study procedures complied with the ethical standards for research with human participants, and was approved by the Institutional Review Board of Chang Gung Medical Foundation (number 201600954B0).

### Data collection and definitions of abnormal lipid profiles

Characteristics data encompass age, gender, dialysis duration, transplant duration, immunotherapy used, body height, weight, body mass index, albumin, estimated glomerular filtration rate, creatinine, low-density lipoprotein cholesterol (LDL-C), high-density lipoprotein cholesterol (HDL-C), triglyceride (TG) and total cholesterol (TC) which were obtained from the participants’ electronic medical records in the same month as the interview.

Lipid profile abnormalities were defined as follows: serum TC levels ≥ 200 mg/dL, serum TG levels ≥ 150 mg/dL, serum LDL-C levels ≥ 100 mg/dL, and serum HDL-C < 40 mg/dL for men and < 50 mg/dL for women; these values were based on the National Cholesterol Education Program Adult Treatment Panel III recommendations^12^.

Dietary data were collected through a 3-day dietary records (by self-reported and including 2 weekdays and 1 day on the weekend) and were assessed by a well-trained registered dietitian during regular followed clinics. Dietary food and nutrient intake were calculated according to Taiwan’s Ministry of Health and Welfare Food and Drug Administration database and analyzed by using CofitPro nutrition analysis software (version 1.0.0, Cofit HealthCare, Taipei, Taiwan), as described previously^6^.

### Scoring method of diet quality indices

To assess diet quality, 3-day dietary data were collected with different indices: AHEI-Taiwan, AHEI-2010, and HEI-2015 (Table 1).

**Table 1:** Clinicodemographic and dietary characteristics of KTRs stratified by the lowest and highest quartiles of AHEI-Taiwan, AHEI-2010, and HEI-2015 scores.

Item	All	AHEI-Taiwan		AHEI-2010		HEI-2015	
		Q1: 26.7–37.7	Q4: 51.3–68.2	Q1: 37.6–55.7	Q4: 68.3–98.8	Q1: 48.4–68.9	Q4: 81.5–89.6
Number, n	102	25	25	26	26	26	26
Age, year	48.9 ± 12.8	42.1 ± 10.7	51.7 ± 14.6*	41 ± 10.4	52.8 ± 13.7^†^	45.6 ± 11.2	50.3 ± 15.7
Male, n (%)	63 (61.7)	18 (72.0)	14 (56.0)	20 (77.0)	14 (53.8)*	18 (69.2)	14 (53.8)
Cadaveric, n (%)	87 (85.3)	19 (76.0)	24 (96.0)	19 (73.1)	22 (84.6)	18 (39.5)	22 (43.7)
Tarcrolimus, n (%)	68 (66.7)	17 (94.4)	14 (100.0)	19 (95.0)	13 (92.9)*	17 (94.4)	13 (92.9)
RT duration, year	8.5 ± 5.8	6.8 ± 4.7	5.8 ± 3.6	7.1 ± 4.4	10.4 ± 5.5*	8.2 ± 6.0	10.5 ± 5.5
Dialysis duration, year	6.6 ± 4.9	0.7 ± 0.5	0.9 ± 0.3	6.6 ± 3.7	5.5 ± 3.9	7.1 ± 6.9	5.0 ± 2.9
Height, cm	162.0 ± 8.6	166.7 ± 8.4	159.1 ± 8.1^†^	166.4 ± 9.0	160.0 ± 8.6*	165.9 ± 8.4	159.2 ± 7.7*
Weight, kg	63.1 ± 13.0	67.2 ± 15.7	61.3 ± 9.7	69.5 ± 14.7	64.2 ± 12.2	68.1 ± 15.5	60.9 ± 9.4
BMI, kg/m^2^	23.9 ± 3.7	24.1 ± 4.7	24.1 ± 3.0	24.9 ± 4.0	24.9 ± 3.3	24.6 ± 4.5	24.0 ± 2.8
TC, mg/dL	205.8 ± 43.9	217.5 ± 38.2	195.6 ± 41.4*	213.5 ± 38.5	203.6 ± 45.2	212 ± 42.5	208.8 ± 50.4
LDL-C, mg/dL	119.8 ± 36.6	134 ± 32.9	108.8 ± 38.6*	130.3 ± 33.6	116.4 ± 36	125.7 ± 28.3	121.2 ± 39.1
HDL-C, mg/dL	52.0 ± 17.9	51.2 ± 16.1	50.4 ± 16.9	53.3 ± 16.8	48.8 ± 16.4	52.7 ± 20.5	48.6 ± 15.6
TG, mg/dL	160.2 ± 121.6	153.7 ± 98	161.4 ± 112.1	149.5 ± 95.7	164.7 ± 86.2	166.2 ± 150	177.6 ± 113.3
Alb, g/dL	4.3 ± 0.3	4.4 ± 0.3	4.3 ± 0.3	4.4 ± 0.3	4.2 ± 0.3*	4.4 ± 0.3	4.3 ± 0.3
Cr, mg/dL	1.5 ± 0.9	1.7 ± 1.0	1.2 ± 0.4^†^	1.8 ± 1.4	1.3 ± 0.7*	1.8 ± 1.0	1.3 ± 0.5^†^
eGFR, ml/min/1.73 m^2^	54.9 ± 20.9	48.5 ± 14.8	64.6 ± 19.7^†^	50.9 ± 18.4	61.4 ± 23.6*	46.1 ± 16.8	61.7 ± 19.0^†^
Energy, kcal	1881.9 ± 367.9	1851.1 ± 353.1	1860.2 ± 340.8	1965.8 ± 325	1831.4 ± 441.5	2046.8 ± 346.5	1752.8 ± 429.7^†^

Data were represented as mean ± SD or n (%) as appropriate.

*AHEI* Alternative Healthy Eating Index, *HEI* Healthy Eating Index, *SD* Standard deviation, *RT* Renal transplant, *BMI* Body mass index, *TC* Total cholesterol, *LDL-C* Low-density lipoprotein cholesterol, *HDL-C* High-density lipoprotein cholesterol, *TG* Triglyceride, *eGFR* Estimated glomerular filtration rate.

*p < 0.05 and ^†^*p* < 0.01.

AHEI-Taiwan is more appropriate for measuring Taiwanese dietary intake and more convenient for calculating the cereal proportion of wholegrain consumption^11^. AHEI-Taiwan scores range from 0 (low diet quality) to 87.5 (high diet quality) and includes nine components: low trans fats; moderate alcohol consumption; high polyunsaturated fatty acid and saturated fatty acid ratio, fruit, vegetable, and wholegrain ratio; white and red meat ratio (white meat was defined as poultry, fish and seafood; red meat was defined as beef, pork and processed meat); nut and soybean intake; and vitamin used. Each component was ranging 0–10 points except vitamin used was ranging 2.5–7.5 points (Table 2).

**Table 2:** Comparison of the lowest and highest quartiles of AHEI-Taiwan scores and components.

Items	Recommendations	All (n = 102)	Q1: 26.7–37.7 (n = 26)	Q4: 51.3–68.2 (n = 26)
Trans fat, %	≤ 1 = 10; ≥ 8 = 0	10.0 ± 0.0	10.0 ± 0.0	10.0 ± 0.0
PSR	≥ 1 = 10; ≤ 0.1 = 0	9.5 ± 1.3	9.0 ± 1.8	9.9 ± 0.4
Fruit, S	2 = 10; 0 = 0	5.1 ± 3.7	1.6 ± 3.0	8.0 ± 2.4^‡^
Vegetable, S	3 = 10; 0 = 0	7.6 ± 2.3	5.9 ± 2.1	8.7 ± 1.9^‡^
Wholegrains ratio	≥ 50% = 10; 0% = 0	1.7 ± 3.4	0.6 ± 1.1	5.0 ± 5.1^‡^
White and red meat ratio	4 = 10; 0 = 0	2.8 ± 2.6	1.7 ± 1.6	4.9 ± 3.5^†^
Nut and soybeans, S	1 = 10; 0 = 0	5.4 ± 4.1	2.6 ± 3.4	7.9 ± 3.4^‡^
Vitamin used, > 5 years	≥ 5 = 7.5; < 5 = 2.5	2.5 ± 0.0	2.5 ± 0.0	2.5 ± 0.0
Alcohol, equivalent	M: 1.5–2.5, F: 0.5–1.5 = 10 M: 0 or > 3.5, F: 0 or > 2.5 = 0	0.1 ± 1.0	0.0 ± 0.0	0.0 ± 0.0
AHEI-Taiwan score	2.5–87.5	44.6 ± 9.0	34.0 ± 2.8	56.9 ± 4.8^‡^

Data are presented as mean ± standard deviation.

*Q* Quartile, *AHEI* Alternative Healthy Eating Index, *S* Servings, *M* Male, *F* Female, *PSR* Polyunsaturated-to-saturated fatty acid ratio.

*p < 0.05; ^†^p < 0.01; ^‡^*p* < 0.001.

AHEI-2010^13^ was modified from AHEI according to the 2015–2020 Dietary Guidelines for Americans, with total scores ranging from 0 (low diet quality) to 110 (high diet quality). AHEI-2010 includes 11 food components: low trans fats, red meat, sodium, and sugar intake with high scores; moderate alcohol consumption with high scores; and high intake of n-3 polyunsaturated fatty acid, fruit, vegetable, wholegrain, and nut and soybean. Each component was ranging 0–10 points (Table 3).

**Table 3:** Comparison of the lowest and highest quartiles of AHEI-2010 scores.

Items	Recommendations	All (n = 102)	Q1: 37.6–55.7 (n = 27)	Q4: 68.3–98.8 (n = 26)
Trans fat, %	≤ 0.5 = 10; ≥ 4 = 0	10.0 ± 0.0	10.0 ± 0.0	10.0 ± 0.0*
n3-PUFA, mg	250 = 10; 0 = 0	8.8 ± 2.2	8.2 ± 2.5	9.5 ± 1.5^†^
PUFA, %	≥ 10 = 10; ≤ 2 = 0	9.7 ± 1.4	9.2 ± 2.1	9.9 ± 0.2^‡^
Fruit, S	4 = 10; 0 = 0	2.9 ± 2.4	1.1 ± 1.6	4.9 ± 2.5^‡^
Vegetable, S	5 = 10; 0 = 0	5.1 ± 2.2	4.0 ± 1.7	6.4 ± 3.1^†^
Wholegrain, S	M: ≥ 90; F: ≥ 75 = 10 0 = 0	1.8 ± 3.1	0.6 ± 1.4	5.1 ± 4.2^†^
Red meat, S	0 = 10; ≥ 1.5 = 0	1.0 ± 2.1	0.0 ± 0.0	2.3 ± 3.1
Nut & soybeans, S	1 = 10; 0 = 0	7.2 ± 4.1	4.0 ± 4.5	9.1 ± 2.4^‡^
Alcohol, equivalent	M: 0–2.5; F:0–1.5 = 10 M: ≥ 3.5, F: ≥ 2.5 = 0	0.2 ± 1.2	0.0 ± 0.0	0.3 ± 1.4*
Sodium, mg	Lowest decile = 10 Highest decile = 0	6.1 ± 3.3	3.7 ± 3.5	8.0 ± 2.0
Sugar, S	0 = 10; ≥ 1 = 0	9.4 ± 0.6	9.1 ± 0.7	9.7 ± 0.4
AHEI-2010 score	0–110	62.1 ± 10.2	50.0 ± 4.5	75.2 ± 6.9^‡^

Data are presented as mean ± standard deviation.

*Q* Quartile, *AHEI* Alternative Healthy Eating Index, *S* Servings, *M* Male, *F* Female, *PUFA* Polyunsaturated fatty acid.

*p < 0.05; ^†^*p* < 0.01; ^‡^*p* < 0.001.

HEI-2015^14^ was developed according to the 2015–2020 Dietary Guidelines for Americans and Diet Pyramid in the United States, with total scores ranging from 0 (low diet quality) to 100 (high diet quality). HEI-2015 has 13 food components: high intake ratio of unsaturated fatty acid and saturated fatty acid; fruit, whole fruit, vegetable, green leaf vegetable, wholegrain, milk, total meat, seafood, and plant with high scores; and low intake of saturated fatty acid, refined grain, sodium, and sugar with high scores. Each component was ranging 0–10 points except fruit, whole fruit, vegetable, green leaf vegetable, total meat, seafood, and plant were ranging 0–5 points (Table 4).

**Table 4:** Comparison of the lowest and highest quartiles of HEI-2015 scores.

Items	Recommendations	All (n = 102)	Q1: 48.4–69 (n = 27)	Q4: 81.5–89.6 (n = 26)
USR	≥ 2.5 = 10; ≤ 1.2 = 0	9.0 ± 1.8	8.2 ± 2.6	9.7 ± 0.7*
SFAa	≤ 8 = 10; ≥ 16 = 0	8.0 ± 2.4	6.6 ± 3.1	8.8 ± 2.0^†^
Fruit^b^	≥ 0.8 = 5; 0 = 0	2.9 ± 2.1	0.6 ± 1.2	4.0 ± 1.5^‡^
Whole fruit^b^	≥ 0.4 = 5; 0 = 0	3.5 ± 2.2	0.9 ± 1.7	4.6 ± 1.4^‡^
Vegetable^b^	≥ 1.1 = 5; 0 = 0	4.3 ± 1.0	3.8 ± 1.3	4.6 ± 0.7*
Green leaf vegetable^b^	≥ 0.2 = 5; 0 = 0	4.7 ± 1.1	4.2 ± 1.6	5.0 ± 0.0^†^
Wholegrain^b^	≥ 3 = 10; 0 = 0	1.8 ± 3.5	0.4 ± 0.9	5.8 ± 5.0^‡^
Refined grain^b^	≤ 3 = 10; ≥ 8 = 0	6.1 ± 3.2	4.8 ± 3.2	8.7 ± 1.9^‡^
Milk^b^	≥ 1.3 = 10; 0 = 0	0.7 ± 1.3	0.3 ± 0.7	1.0 ± 1.2
Total meat^b^	≥ 2.5 = 5; 0 = 0	4.7 ± 0.7	4.6 ± 0.9	4.8 ± 0.8
Seafood and plant^b^	≥ 0.8 = 5; 0 = 0	5.0 ± 0.3	5.0 ± 0.0	4.9 ± 0.7
Sodium^c^	≤ 1.1 = 10; ≥ 2 = 0	8.3 ± 3.0	5.5 ± 4.0	10.0 ± 0.2^‡^
Sugar^b^	≤ 6.5 = 10; ≥ 26 = 0	10.0 ± 0.4	9.9 ± 0.6	10.0 ± 0.3
HEI-2015 scores	0–100	69.1 ± 11.0	54.9 ± 5.1	82.5 ± 6.4^‡^

Data are presented as mean ± standard deviation.

*USR* Unsaturated and saturated fatty acid ratio, *SFA* Saturated fatty acid.

^a^Percentage of energy.

^b^Servings/1000 kcal.

^c^gram/1000 kcal.

*p < 0.05; ^†^*p* < 0.01; ^‡^*p* < 0.001.

### Statistical analysis

SAS 9.4 version software (SAS Institute, Cary, NC, USA) was used for statistical analyses. Data are presented as percentage, interquartile range, mean and standard deviation, as appropriate. The associations between diet quality and lipid profile abnormalities as risk factors for CVD by logistic regression analysis (adjusted for age, gender, energy intake, transplant and dialysis duration, and Charlson comorbidity index) based on the Kidney Disease Outcomes Quality Initiative (KDOQI) guidelines^15^. Data are described as odds ratios (ORs) with 95% confidence intervals (95% CIs) and *p* value < 0.05 was significance.

## Results

### Baseline characteristics and comparisons between the lowest and highest quartiles of dietary indices

**Figure 1:**
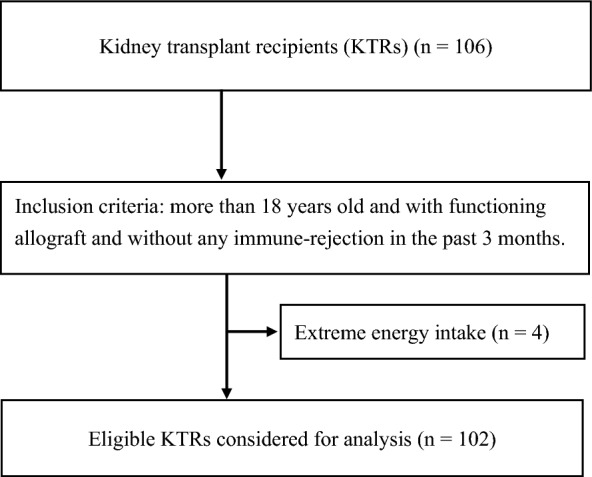
Patient recruitment flowchart.

We enrolled 102 eligible KTRs (Fig. 1). The mean serum albumin level and estimated glomerular filtration rate (eGFR) were 4.3 ± 0.3 g/dL and 54.9 ± 20.9 mL/min/1.73 m^2^, respectively. KTRs exhibited adequate dietary intake and graft function, which was in chronic kidney disease stage 3A based on the KDOQI guidelines (15). The mean LDL-C, HDL-C, TC, and TG (as risk factors for CVD) were 119.8 ± 36.6, 52.0 ± 17.9, 205.8 ± 43.9, and 160.2 ± 121.6, respectively. The numbers and percentages of participants with abnormal TC, LDL-C, HDL-C, and TG levels were 52 (50.9%), 35 (34.3%), 36 (35.3%), and 32 (31.4%), respectively. Ninety-two KTRs had at least one risk factor for CVD.

Compared with the lowest quartile, the highest quartile of AHEI-Taiwan had lower TC and LDL-C levels, whereas lipid profiles were not significantly different between the lowest and highest quartiles of AHEI-2010 and HEI-2015. (Table 1).

### Comparison of the lowest and highest quartiles of AHEI-Taiwan scores

Most KTRs had inadequate wholegrain ratio, lower white and red meat ratio, and lower alcohol consumption, with the scores being less than half of the corresponding scores recommended in AHEI-Taiwan.

Compared with the lowest quartiles, the highest quartiles of AHEI-Taiwan had significantly higher total scores and scores for fruits, vegetables, ratio of wholegrains, white and red meat ratio, and nut and soybean.

### Comparison of the lowest and highest quartiles of AHEI-2010 scores

Most KTRs had inadequate wholegrain ratio, excessive red meat intake, and low alcohol consumption, with the scores being less than half of the corresponding scores recommended in AHEI-2010.

Compared with the lowest quartiles, the highest quartiles of AHEI-2010 had significantly higher total scores and scores for trans fats, n-3 PUFAs, fruits, vegetables, whole grains, nuts and soybeans, and alcohol consumption.

### Comparison of the lowest and highest quartiles of HEI-2015 scores

Most KTRs had inadequate wholegrain and milk consumption, with the scores being less than half of the corresponding scores recommended in HEI-2015. Compared with the lowest quartiles, the highest quartiles of HEI-2015 had significantly higher total scores and scores for the ratio of unsaturated and saturated fatty acid, saturated fatty acid, fruit, whole fruit, vegetable, green leaf vegetable, wholegrain, refined grain, and sodium consumption.

### OR of dietary indices and CVD risk factors

Logistic regression analysis is presented in Table 5. Model 2 was adjusted for age, gender, energy intake, Charlson comorbidity index, transplant duration, and dialysis duration based on the KDOQI guidelines (15). The highest quartiles of AHEI-Taiwan had 82% (OR 0.18; 95% CI 0.04–0.72, *p* for trend < 0.05) lower odds of high TC levels and 88% (OR 0.12; 95% CI 0.03–0.58, *p* for trend < 0.05) lower odds of high LDL-C levels. No significant association was observed between the lipid profile and AHEI-2010 scores. However, the highest quartiles of HEI-2015 had 81% (OR 0.19; 95% CI 0.04–0.83, *p* for trend < 0.05) lower odds of high LDL-C levels.

**Table 5:** Odds ratio of dietary indices and cardiovascular disease risk factors.

Items	AHEI-Taiwan				AHEI-2010				HEI-2015			
	Q1	Q2	Q3	Q4	Q1	Q2	Q3	Q4	Q1	Q2	Q3	Q4
TC												
Crude	1 (ref)	0.25 (0.08–0.80)	0.75 (0.24–2.39)	0.31 (0.10–1.00 )	1 (ref)	0.29 (0.09–0.93)*	1.11 (0.36–3.46)	0.54 (0.18–1.62)	1 (ref)	0.30 (0.09–0.94)	0.52 (− .17–1.61)*	0.77 (0.25–2.37)
Model 1	1 (ref)	0.17 (0.05–0.59)*	0.51 (0.14–1.79)	0.20 (0.05–0.72)*	1 (ref)	0.14 (0.04–0.55)^†^	0.70 (0.20–2.42)	0.30 (0.08–1.05)	1 (ref)	0.24 (0.07–0.78)	0.33 (1.00–1.16)	0.63 (0.20–2.04)
Model 2	1 (ref)	0.18 (0.04–0.73)*	0.61 (0.16–2.36)	0.18 (0.04–0.77)*	1 (ref)	0.19 (0.05–0.78)*	0.96 (0.24–3.85)	0.29 (0.08–1.148)	1 (ref)	0.25 ( 0.07–0.92)	0.39 ( 0.11–1.45)	0.50 ( 0.14–1.80)
LDL-C												
Crude	1 (ref)	0.19 (0.05–0.71)*	0.52 (0.13–2.05)	0.21 (0.06–0.78)*	1 (ref)	0.67 (0.21–2.12)	0.79 (0.25–2.54)	0.84 (0.26–2.68)	1 (ref)	0.31 (0.08–1.15)	0.24 (0.06–0.92)*	0.26 (0.07–0.98)*
Model 1	1 (ref)	0.12 (0.03–0.52)^†^	0.28 (0.06–1.27)	0.11 (0.03–0.50)^†^	1 (ref)	0.49 (0.14–1.79)	0.52 (0.15–1.84)	0.54 (0.15–1.95)	1 (ref)	0.25 (0.06–0.99)*	0.15 (0.04–0.66)*	0.21 (0.05–0.85)*
Model 2	1 (ref)	0.18 (0.04–0.81)*	0.32 (0.07–1.52)	0.12 (0.03–0.58)*	1 (ref)	0.80 (0.20–3.14)	0.48 (0.12–1.89)	0.58 (0.15–2.26)	1 (ref)	0.26 (0.06–1.09)	0.16 (0.04–0.74)*	0.19 ( 0.04–0.83)*
HDL-C												
Crude	1 (ref)	2.94 (0.91–9.46)	2.05 (0.66–6.31)	1.63 (0.53–4.98)	1 (ref)	18.4 (3.55–95.5)^‡^	2.04 (0.67–6.22)	2.56 (0.84–7.83)	1 (ref)	3.61 (1.08–12.03)*	2.31 (0.73–7.27)	1.26 (0.41–3.80)
Model 1	1 (ref)	2.65 (0.54–12.96)	2.1 (0.44–10.05)	0.88 (0.16–4.74)	1 (ref)	12.86 (1.85–89.21)^†^	1.43 (0.27–7.54)	1.50 (0.30–7.57)	1 (ref)	3.64 (0.84–15.74)	0.62 (0.10–4.02)	0.54 (0.11–2.69)
Model 2	1 (ref)	4.09 (0.63–26.34)	3.26 (0.51–20.95)	0.65 (0.1–4.31)	1 (ref)	48.16 (3.04–76.25)^†^	0.61 (0.08–4.59)	0.64 (0.10–4.14)	1 (ref)	4.26 (0.86–21.3)	0.53 (0.07–3.96)	0.56 (0.10–3.08)
TG												
Crude	1 (ref)	0.61 (0.17–2.27)	1.36 (0.41–4.47)	1.71 (0.53–5.60)	1 (ref)	1.05 (0.29–3.84)	1.3 (0.37–4.58)	2.86 (0.87–9.43)	1 (ref)	0.52 (0.13–2.05)	1.53 (0.46–5.02)	1.99 (0.62–6.38)
Model 1	1 (ref)	0.62 (0.16–2.38)	1.33 (0.38–4.67)	1.71 (0.5–5.94)	1 (ref)	1.19 (0.30–4.77)	1.37 (0.36–5.19)	3.08 (0.85–11.18)	1 (ref)	0.79 (0.22–2.83)	1.25 (0.35–4.51)	1.65 (0.50–5.46)
Model 2	1 (ref)	0.45 (0.11–1.86)	0.97 (0.26–3.62)	1.21 (0.33–4.45)	1 (ref)	1.08 (0.26–4.56)	1.17 (0.29–4.80)	2.55 (0.67–9.71)	1 (ref)	0.80 (0.21–2.98)	1.23( 0.33–4.63)	1.61 (0.46–5.67)

Data were represented as odds ratio and 95% confidence interval. Model 1 adjusted for age and gender. Model 2 adjusted for age, gender, energy intake, renal transplant and dialysis duration, and Charlson comorbidity index.

*Q* Quartile, *OR* Odds ratio, *CVD* Cardiovascular disease, *CI* Confidence interval, *AHEI* Alternative Health Eating Index, *TC* Total cholesterol, *LDL-C* Low-density lipoprotein cholesterol, *HDL-C* High-density lipoprotein cholesterol, *TG* Triglyceride.

*p < 0.05; ^†^*p* < 0.01.

## Discussion

Our results demonstrated that KTRs in the highest quartiles of AHEI-Taiwan had an 82% and 88% lower odds of high TC and high LDL-C levels, respectively. Moreover, the highest quartiles of HEI-2015 had 77% lower odds of high LDL-C levels than the lowest quartiles of these dietary indices after adjustment for age, gender, energy intake, transplant and dialysis duration, and Charlson comorbidity index.

CVD remains one of the leading causes of KTR mortality and increases graft function loss^2^. High diet quality represents healthy dietary guidance from dietary indices and is associated with lower risk of all-cause, cancer, and mortality^16^. In the present study, the mean total HEI-2015 and AHEI-2010 scores were 69.1 and 62.1, which were higher than those reported in some countries: 45.7 in a Brazilian population^17^; 42.2 and 43.8 in Chinese male and female populations, respectively^18^; and 52.4 and 47.6 in U.S. male and female populations, respectively^13^. High diet quality, as measured using dietary indices based on foods, nutrients and dietary patterns, is associated with a low risk of chronic disease^19^. In the Women’s Health Initiative Observational Study^20^, which included postmenopausal women cohort study, demonstrated that the highest quintile of AHEI score had a 23% reduction in the risk of CVD (HR, 0.77; 95% CI 0.70–0.84) and a 30% reduction in the risk of heart failure (HR, 0.70; 95% CI 0.59–0.82) compared with the lowest quintile. Consistent with the results of a prospective analysis of U.S. male health professionals^21^, the highest quintile of AHEI scores had an 11%–20% lower risk of major chronic disease (CVD, cancer, or death) as well as a 28%–39% education in CVD risk compared with lowest quintile. However, over 24 years of follow-up, the highest quintile of the AHEI-2010 scores (13) also had a significantly lower risk of CVD (24%), diabetes (33%), CHD (31%), stroke (20%), and major chronic disease risk (19%) than the lowest quintile. A recent review^22^ also concluded that higher diet quality for AHEI was associated with a lower incidence of all-cause mortality and CVD mortality; higher diet quality for HEI also associated with a lower risk of CVD mortality.

Previous study has been demonstrated that participants with higher AHEI scores had lower LDL-C and TG levels^23^. Kauffman et al.^10^ also indicated that a higher AHEI score was associated with lower serum LDL-C and TC levels. Consistently, our data indicated that KTRs with higher AHEI-Taiwan scores had 82% and 88% lower odds of high TC and high LDL-C levels, respectively, but TG was not significantly different. No significant difference in lipid profile parameters was noted between the highest and lowest quartiles of AHEI-2010. Similarly, Ziaee et al.^8^ demonstrated that higher HEI scores were related to lower LDL-C levels among 235 participants. Another study found that higher meat and sweetened beverage intake was associated with higher levels of LDL-C, TC, TG, and lower levels of HDL-C^24^.

The possible mechanism of high diet quality had a lower risk of lipid profile abnormalities is related to the AHEI-2010, AHEI-Taiwan, and HEI-2015 guidelines, which emphasize a high polyunsaturated fatty acids intake in the form of nuts and soybeans, because their anti-inflammatory properties prevent atherosclerosis^25^. Wholegrain foods are a rich source of dietary fiber that binds cholesterol and bile acids in the intestinal lumen to decrease serum TC and LDL-C levels^26^. They can also enhance the cholesterol-lowering effect of statins^27^. Fruits and vegetables are rich sources of fiber, antioxidants, and polyphenols, which decrease serum TC and LDL-C levels^28^, prevent the oxidation of cholesterol in the arteries^29^, and decrease systemic inflammation through cell signaling processes, thus preventing atherosclerosis and CVD development^30^. An intervention study^31^ concluded that consuming three servings of fruit and two servings of vegetables every day for 4 weeks significantly decreased TC by 15.29 mg/dL and LDL-C by 10.45 mg/dL in line with the recommendations of AHEIT-Taiwan regarding vegetable and fruit consumption^11^.

Red meat is rich in saturated fatty acids, which increases LDL-C levels by enhancing apolipoprotein B-containing lipoprotein production and inhibiting LDL receptor activity^32^. Substituting saturated fats with polyunsaturated fat as cooking oil reduces LDL-C levels and the TC to HDL-C ratio, which is beneficial for coronary heart disease prevention^33^. Alcohol consumption was reported to be positively associated with TG levels and inversely associated with HDL-C levels^34^. Another study noted that high alcohol consumption caused significantly increased LDL-C, TC, and TG levels and decreased the levels of HDL-C^35^. By contrast, moderate alcohol consumption seems to have a protective effect on the heart. Taken together, the aforementioned evidence supports that a healthy dietary index inclusive of high polyunsaturated fatty acids, vegetables, whole grains, fruits, and less saturated fatty acids and red meat consumption may reduce the risk of lipid disorders. AHEI-Taiwan was modified from AHEI according to Taiwanese dietary recommendations^11^ which is more adapted to Taiwanese dietary patterns and has a more protective effect on abdominal lipid profiles in KTRs.

Few studies have evaluated the association between diet quality and metabolic disorders in KTRs, especially in Taiwan. A healthy diet can minimize the risk of lipid profile abnormalities, thus providing protection against CVDs, improving quality of life, and extending the graft kidney survival rate.

This study has some limitations. First, the cross-sectional design precluded the determination of causality although we used a 24-h recall method collected a 3-day dietary records to increase the precision of nutritional assessment. Future well-designed randomized controlled trials should assess whether our observations can be extrapolated to other KTRs. Second, different assessment methods for dietary food and nutrients intake and determining diet quality indices may have contributed to inconsistent findings. Further development of validated diet quality indices as a dietary assessment tool is extremely desirable for increasing clinician assessment efficiency to promote healthy diet education. Finally, this study’s findings may remain be restricted by other potential or unmeasured confounding factors, such as immunological therapy or family history. However, our findings focus attention on a better diet quality which is an important affecting factor was associated with the preventions of lipid profile abnormalities.

## Conclusion

This prospective study demonstrated that higher adherence to healthy diet quality, such as AHEI-Taiwan and HEI-2015, was associated with lower lipid profile abnormalities as risk factors for CVD in KTRs. Notably, AHEI-Taiwan is developed according to Taiwan’s dietary recommendation which is more closely to Taiwanese dietary culture. Further study regarding to diet quality and the education strategy of health promoting to prevent the abnormalities lipid profiles are warranted for long-term KTRs.

## Data Availability

The datasets used and/or analyzed during the current study available from the corresponding author on reasonable request.
